# *Elizabethkingia meningosepticum* in a Patient with Six-Year Bilateral Perma-Catheters

**DOI:** 10.1155/2014/985306

**Published:** 2014-08-05

**Authors:** Konstantin Boroda, Li Li

**Affiliations:** Nassau University Medical Center, 2201 Hempstead Tpke, East Meadow, NY 11554, USA

## Abstract

*Elizabethkingia meningosepticum* (EM) is a saprophyte which is ubiquitous in nature, but not normally present in the human flora. Instances of infection are rare in the USA, but EM may be an emerging pathogen among immune-compromised patients. EM can cause a variety of infections, but nosocomial pneumonia and bacteremia have been the most commonly reported among immune-compromised adults. EM has proven difficult to treat with a mortality rate of 23%–41% in adult bacteremia. This is likely due to its resistance to commonly used empiric antibiotics for Gram-negative infections. A review of the literature suggests that there has been a shift EM's susceptibility profile over time along with a great variability in antibiotic susceptibilities reported. This signifies the importance of close monitoring of these changes. In this report we present a case of a 64-year-old male with end stage renal disease and bilateral subclavian perma-catheters, who was admitted with systemic inflammatory response syndrome. While initial peripheral blood cultures were negative, cultures later drawn from his perma-catheters revealed *Corneybacterium* species and EM. The patient was initially treated with empiric vancomycin and piperacillin-tazobactam. After antibiotics susceptibilities became available, he was treated with levofloxacin and ceftazidime. The patient improved, was culture negative, and later had perma-catheter removal.

## 1. Introduction 

*Elizabethkingia meningosepticum* (EM) is nonfermentative, nonmotile, oxidase-positive, Gram-negative rod that is ubiquitous in nature; it can be found in soil, plants, and water sources, including those in hospitals; it is not normally present in human flora [1]. This organism was first identified in 1959 by King, the bacterium was unclassified, and at that time it was named* Flavobacterium meningosepticum*. The genus was then reclassified under the* Chryseobacterium *genus in 1994. Then in 2005 it was proposed to be renamed under the* Elizabethkingia *genus based on 16 S rRNA genomic sequence similarity [2]. EM infection is uncommon worldwide; however, rates of reported cases in the intensive care unit setting have been rising over recent years [3, 4]. Instances of infection are rare in the USA, but EM may be an emerging pathogen among immunocompromised patients in this region. We present a case of EM identified in a patient with long-term perma-catheter placement for hemodialysis.

## 2. Case Presentation

A 64-year-old African American man with polycystic kidney disease, hypertension, Hepatitis C, and end stage renal disease was admitted with nausea, chills, and four episodes of nonbloody emesis during hemodialysis. On presentation, the patient was febrile to 103°F and tachycardic. Physical examination was significant for tachypnea, diaphoresis, and bilateral subclavian perma-catheters, which had been in place for 6 years. Blood cultures were drawn on and empiric vancomycin and piperacillin-tazobactam were started. He initially had a normal Leukocyte count of 4.97 (1000 cells/mL), which drastically increased to 22.55 (1000 cells/mL), with a bandemia; on the morning of the second day of admission, he received one dose of gentamicin at that time. On day 2 and day 3, the patient remained hemodynamically stable and afebrile. His leukocyte count trended down, but still remained elevated. His blood cultures remained negative. The patient was dialyzed on day 4, during which blood cultures were drawn from his perma-catheters; immediately after his dialysis session the patient signed out against medical advice. He was given oral Augmentin on discharge. Gram stain from the second set of cultures revealed gram variable rods and the patient was brought to the emergency department from outpatient hemodialysis again. Upon his second admission, the patient was afebrile and leukocytosis was completely resolved. He received vancomycin and one dose of gentamycin. The blood culture, which was drawn from his perma-catheters, later grew* Corynebacterium *species and* E. meningosepticum*. The isolated EM was susceptible to cefotaxime, ceftazidime, levofloxacin, tetracycline, and trimethoprim-sulfamethoxazole; it was resistant to ciprofloxacin. His antibiotic regimen was switched to ceftazidime and levofloxacin based on these results. Upon positive blood cultures, his perma-catheters were planned to be removed and he was dialyzed via femoral Shiley catheter. The patient remained stable on the internal medicine unit during his second admission. Repeated blood culture from one of his perma-catheters was negative. The patient completed 4 days of antibiotics during his first admission and 10 days during his second. His hospital course included a complicated attempt at removing his indwelling catheters due to scar tissue formation, but they were removed and he later underwent arteriovenous fistula placement as outpatient.

## 3. Discussion 

EM is a rare cause of illness, but it may be an emerging pathogen in the USA among certain populations. Historically, EM has been known to cause meningitis in premature and newborn infants; in the nonneonatal population, it affects immunocompromised adults. EM has been reported to cause a variety of infections in immunocompromised adults including pneumonia, bacteremia, sepsis, endocarditis, and meningitis [1]. Additionally, there have been reports of osteomyelitis, abdominal abscesses, ocular infection, sinusitis, bronchitis, epididymitis, dialysis associated peritonitis, and prosthesis associated joint infection [5]. Several recent studies report that primary bacteremia is the most common type of infection among the immunocompromised adult population, with most reports and outbreaks occurring in Taiwan and Southeast Asia [3, 4].

EM infection is mostly spread through nosocomial means; it has been thought to enter hospitals through community water supplies as it can survive in chlorine-treated water sources [6]. EM has been found in saline solutions, disinfectants, humidifiers, respirators, and the hands of hospital staff [5]. Colonization of implantable medical devices like intravascular catheters has also been reported and was the most likely cause of infection in the patient of the present case [6].

EM has proven to be very difficult to treat, with high rates of mortality in immunocompromised patients [1]. Healthcare associated EM infection has been reported to have a 28-day mortality of 41%, while community acquired infection has a 9.1% mortality rate [3]. Treatment failure may result from a delay in the identification of the organism and its susceptibilities. Discrepancies exist between the antibiotic susceptibility testing methods of disk-diffusion and broth dilution, with broth dilution being shown as more reliable in determining susceptibility [1, 7]. In addition, EM has been reported to have an unusual and, at this time, unpredictable, antibiotic susceptibility pattern.

Even though EM is classified as a Gram-negative organism, it is surprisingly resistant to antibiotics commonly used to treat Gram-negative bacteria like Beta-lactams, carbapenems, aminoglycosides, and chloramphenicol, while it is susceptible to agents which are used to treat Gram-positive bacterial infections including Fluoroquinolones, trimethoprim-sulfamethoxazole, vancomycin, and rifampin [1, 3–6, 8, 9]. These peculiar characteristics make the antibiotic susceptibility pattern unpredictable and the choice of antibiotic therapy difficult.

Vancomycin has been suggested as the empiric therapy of choice in reports from the 1960's to 1980's [1]. However, recent research indicates increasing resistance, likely acquired due to widespread use of vancomycin in immunocompromised patients.  Table 1 outlines the range of susceptibility to various antibiotics noted in several publications. As seen in Table 1, studies had isolates with a wide range of susceptibility to vancomycin [1, 3, 4, 6].

Furthermore, geographically based variation in antibiotic susceptibility may exist. Kirby et al. found that 100% of North American and European isolates were susceptible to piperacillin-tazobactam, with only 50% of isolates susceptible in Southeast Asia [6]. EM isolates in Asia were also more resistant to cephalosporins than isolates from North America and Europe [4]. This variation is in part related to production of extended spectrum beta-lactamases in some strains [10].

Additionally, as seen in Table 1, trimethoprim-sulfamethoxazole and fluoroquinolones displayed superb* in vitro* activity against EM infection and several authors recommend them for empiric therapy [1, 3, 4, 6, 10].

On the contrary, several antibiotics have poor* in vitro* susceptibility. These include aminoglycosides, colistin, and carbapenems. Aminoglycosides had ranges of 0–8% susceptibility in prior reviews [1, 3, 6]. Similarly, carbapenems had poor activity against EM in these reports. Carbapenem use has been associated with higher rate of 14-day mortality in EM-infected patients [4, 10].

In our present case report, our EM bacterial isolate was susceptible to cefotaxime, ceftazidime, levofloxacin, tetracycline, and trimethoprim-sulfamethoxazole, and it was resistant to ciprofloxacin. Our patient had rapid clinical improvement on vancomycin and piperacillin-tazobactam. It is reasonable to conclude that this strain of EM may have been susceptible to piperacillin-tazobactam, based on the high rates of susceptibility identified in the recent literature [3, 4].

Considering EM's evolving and unusual susceptibility profile, future research should focus on close surveillance of these changes. Additionally, development of rapid microbial identification and sensitivity testing techniques will assist with the selection of appropriate and effective antibiotics for such atypical bacterial infections.

## Figures and Tables

**Table 1:** Ranges of percentage of EM isolates susceptible to each antibiotic from 4 long-term clinical investigations [1, 3, 4, 6, 10].

Antibiotics	Ranges of	References
	susceptible isolates (%)	
Ciprofloxacin	43.4–91.7	[1, 3, 4, 6]
Levofloxacin	62.5–81.8	[1, 3, 4, 6, 10]
Gatifloxacin	65.4–100	[6, 8]
Garenoxacin	100	[6]
Moxifloxacin	87.9	[4]
Piperacillin tazobactam	11.5–90.6	[3, 4, 6, 10]
Ticarcillin-clavulanate	78.8	[4]
Trimethoprim sulfamethoxazole	65–96.9	[1, 3, 4, 6]
Rifampin	69.2–93	[1, 6, 10]
Cefepime	0–100	[3, 4, 6]
Ceftazidime	0–34.6	[3, 4, 6, 10]
Aztreonam	0–11.5	[3, 10]
Imipenem	0–34.6	[3, 4, 6, 10]
Meropenem	0–34.6	[4, 6, 10]
Vancomycin	0–65	[1, 3, 4, 6]
Amikacin	0–21.2	[3, 4, 6]
Gentamicin	16.7–18.2	[4, 6]
Colistin	0	[3, 4]
Tigecycline	32.3–84.4	[3, 4]
Doxycycline	78.8	[4]
